# Application of Pharmacokinetic and Pharmacodynamic Analysis to the Development of Liposomal Formulations for Oncology

**DOI:** 10.3390/pharmaceutics6010137

**Published:** 2014-03-18

**Authors:** Sihem Ait-Oudhia, Donald E. Mager, Robert M. Straubinger

**Affiliations:** 1 Department of Pharmaceutical Sciences, University at Buffalo, State University of New York, Amherst, NY 14214, USA; E-Mails: dmager@buffalo.edu (D.E.M.); rms@buffalo.edu (R.M.S.); 2 Departments of Cancer Pharmacology and Therapeutics, and Molecular and Cellular Biophysics and Biochemistry, Roswell Park Cancer Institute, Elm/Carton Streets, Buffalo, NY 14263, USA

**Keywords:** liposomes, nanoparticulate drug carriers, drug delivery, pharmacokinetics, pharmacodynamics, mathematical simulation

## Abstract

Liposomal formulations of anticancer agents have been developed to prolong drug circulating lifetime, enhance anti-tumor efficacy by increasing tumor drug deposition, and reduce drug toxicity by avoiding critical normal tissues. Despite the clinical approval of numerous liposome-based chemotherapeutics, challenges remain in the development and clinical deployment of micro- and nano-particulate formulations, as well as combining these novel agents with conventional drugs and standard-of-care therapies. Factors requiring optimization include control of drug biodistribution, release rates of the encapsulated drug, and uptake by target cells. Quantitative mathematical modeling of formulation performance can provide an important tool for understanding drug transport, uptake, and disposition processes, as well as their role in therapeutic outcomes. This review identifies several relevant pharmacokinetic/pharmacodynamic models that incorporate key physical, biochemical, and physiological processes involved in delivery of oncology drugs by liposomal formulations. They capture observed data, lend insight into factors determining overall antitumor response, and in some cases, predict conditions for optimizing chemotherapy combinations that include nanoparticulate drug carriers.

## 1. Introduction

In the 1960s it was observed that purified phospholipids from biological sources spontaneously form closed vesicular structures when hydrated. Since that seminal discovery, a sustained effort has been devoted to the development of lipid vesicles (liposomes) as a drug delivery system for numerous applications, including cancer therapy [1,2]. Structurally, liposomes are microscopic vesicles that enclose an internal aqueous space within one or more bilayer membranes composed of natural or synthetic phospholipids. They are biocompatible and biodegradable, generally low in toxicity, and seldom immunogenic. A variety of approaches permit control of particle size, and the smallest liposomes qualify as nanoparticulate drug carriers.

**Figure 1:**
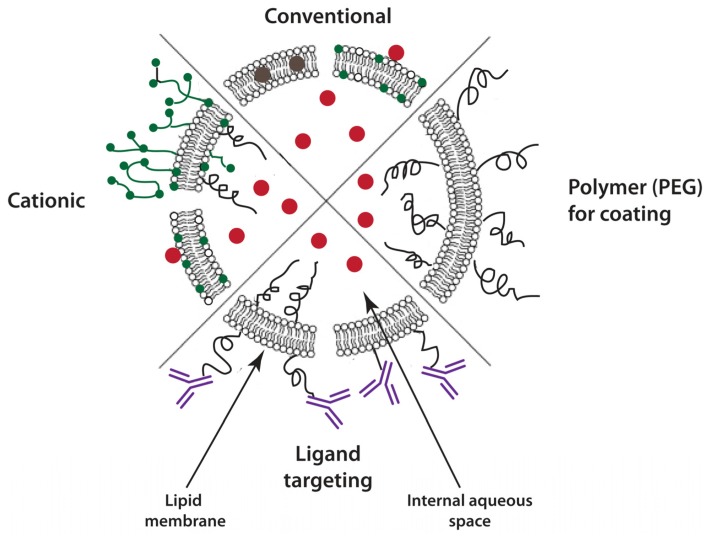
Schematic representation of four categories of drug-loaded liposomes. The liposome consists of a bilayer phospholipid membrane surrounding an internal aqueous core. Drugs may be incorporated in either compartment, depending on their partition coefficient. The surface of the prototype liposome is unmodified, and its charge reflects the molar ratio of neutral or negatively charged phospholipids. The bilayer may be modified to display (i) polymers such as polyethylene glycol (PEG) on the surface to prolong the plasma circulation time (sterically stabilized liposomes); (ii) immunoglobulins or immunoglobulin fragment antigen binding (Fab’) to target specific antigens or receptors (immunoliposomes); or (iii) positive charge (cationic liposomes). Adapted with permission from [25]. Copyright 2014 Elsevier.

Figure 1 depicts schematically the numerous physicochemical characteristics and modifications that have been employed to optimize the liposome carrier for different applications [3,4]. The limiting phospholipid bilayer membrane surrounds an internal aqueous space that can be used to encapsulate hydrophilic chemotherapeutic drugs, whereas hydrophobic agents can be accommodated by incorporation into the membrane [3,5]. The bilayer membrane can include components that provide physicochemical control over pharmacokinetic (PK) properties such as elimination half-life, biodistribution, permeability, and drug release rate. In addition to the membrane physicochemical properties that affect drug release rate and PK, which will be discussed below, the external surface of the liposome can be modified in several ways that can alter biodistribution significantly: (i) glycolipids or synthetic hydrophilic polymers such as polyethylene glycol (PEG) covalently bound to the membrane can produce sterically stabilized liposomes (SSL), which have reduced opsonization and extended circulating plasma half-life; (ii) the surface can be decorated covalently with targeting ligands that enhance binding and internalization by cancer cells expressing a receptor for the ligand, such as immunoglobulins or their subunits (e.g., immunoglobulin fragment antigen binding (Fab’); and single-chain variable fragments, scFv), or nutrient molecules having affinity for cell surface receptors (e.g., transferrin; folate); and (iii) engineered for triggered release of the encapsulated drug at the tumor site, which can be achieved by sensitizing the bilayer membrane to a specific stimuli such pH, light, oxidation, enzymatic degradation, heat, or radiation [6,7]. As an example, through mild hyperthermia (41 °C), liposomal doxorubicin (L-DXR) showed an improvement in the tumor vasculature permeability, a subsequent increase in liposome extravasation, and an enhanced interstitial penetration [8,9].

Numerous liposome-based products have been approved or marketed [10]. For oncology applications, several classes of antineoplastic agents have been incorporated into liposomes in order to increase their therapeutic index. They include taxanes [11,12,13], anthracyclines [14,15], platinum analogs [16,17], camptothecins [18,19,20,21], *Vinca* alkaloids [22,23], and antimetabolites [24]. Numerous liposomal anticancer drugs are in late stage clinical development or are clinically approved. A selection is shown in Table 1. Food and Drug Administration (FDA) approved products include conventional- and SSL-based formulations of doxorubicin (Myocet^®^ and Doxil^®^), daunorubicin (DaunoXome^®^), vincristine (Marqibo^®^), and cytarabine (DepoCyte^®^).

In some cases, liposome incorporation can increase the antitumor efficacy of the encapsulated drugs by providing more selective delivery or targeting to the tumor tissue, whereas in other cases, toxicity is reduced by avoidance of critical normal tissues. Ultimately, modulation of the pharmacokinetic and pharmacodynamic (PD) properties of cytotoxic drugs is responsible for improving their overall pharmacological properties. Examples of beneficial pharmacokinetic effects mediated by liposomes include: reducing metabolism or inactivation of labile drugs in the plasma or tissues; extending drug circulating half-life by reducing drug removal (clearance) from the blood; decreasing drug distribution to healthy tissues because of the particle size limitation for transport across healthy vascular endothelium; and increasing the fraction of the injected dose delivered to the tumor site because of flaws in the tumor vascular endothelium, which underlies the extended permeability and retention (EPR) of nanoparticulates in the tumor interstitium [3,26]. Examples of beneficial pharmacodynamic effects mediated by liposomes are more elusive to identify; alterations of PD would manifest themselves following careful analysis and accounting for PK effects on drug biodistribution at the organismal, tissue, and cellular levels. It is likely that the source of most apparent changes in PD, such as an increase in potency of an encapsulated drug relative to the unencapsulated drug, actually arise from biodistributional (PK) effects such as demonstrated for the gemcitabine-loaded innovative nanocarrier formulation [27]

**Table 1:** Available liposomal drugs in oncology.

Approved liposomal anticancer chemotherapeutics			
**Liposomal anticancer drug**	**Brand name**	**Indications**	**References**
Pegylated liposomal DXR	Doxil^®^	AIDS-related Kaposi’s sarcoma	[28,29,30]
		Metastatic ovarian cancer	
		Metastatic breast cancer	[31,32,33,34]
		Multiple myeloma	[35,36,37,38]
Non-pegylated liposomal DXR	Myocet^®^	Same indications as Doxil^®^	[39,40,41]
Liposomal daunorubicin	DaunoXome^®^	AIDS-related Kaposi’s sarcoma	[42]
Liposomal cytarabine		Acute myeloid leukemia	[43]
	DepoCyte^®^	Lymphomas and leukemia with meningeal spread	[44]
**Liposomal anticancer drugs in development**			
**Drug name**	**Encapsulated drug**	**Stage of development**	**References**
Liposomal annamycin	Annamycin	Phase II	[45,46]
SPI-77	Cisplatin	Phase II	[16,47,48,49]
Lipoplatin	Cisplatin	Phase III	[50]
LiPlaCis	Cisplatin	Phase I	[51]
l-NDDP/aroplatin	Cisplatin analogue	Phase II	[17,52]
ThermoDox^®^	Doxorubicin	Phase II	[53]
JNS002	Doxorubicin	Phase II	[54]
TLI	Topotecan	Trial	www.clinicaltrials.gov
OSI211	Lurtotecan	Phase III	[52,55]
LEM	Mitoxantrone	Preclinical	[56]
NL CPT-11	Camptothecin	Trial	www.clinicaltrials.gov
L9NC	9-Nitro-20-( *S*)-camptothecin	Trial	www.clinicaltrials.gov
PNU-93914	Paclitaxel	Trial	www.clinicaltrials.gov
LEP-ETU	Paclitaxel	Trial	www.clinicaltrials.gov
IHL-305	Irinotecan	Phase I	[57]
PEP02	Irinotecan	Phase I	[58]
MBP426	Oxaliplatin	Phase I	[59]
LE-SN38	Active metabolite of Irinotecan	Trial	www.clinicaltrials.gov
Marqibo^®^	Vinscristine	Phase II	[60]
VLI	Vinorelbine	Trial	www.clinicaltrials.gov
CPX-1	Combination: Irinotecan + Floxuridine	Phase I	[61]
CPX-351	Combination: Cytarabine + Daunorubicin	Phase I	[62]

The development of liposome-based formulations involves optimization of a considerable number of parameters that ultimately impact therapeutic performance. Although many guiding principles are known, optimization remains largely empirical. A quantitative basis by which to rationalize or anticipate the impact of changes in the myriad factors controlling therapeutic efficacy is almost entirely lacking. New strategies are required to overcome these challenges; one consists of developing *in silico* approaches, such as mathematical modeling, to identify experimentally testable hypotheses that explain how carrier-based formulations can improve PK characteristics, and describe quantitatively the pharmacological action of carrier-associated anticancer drugs. Advanced PK and PD modeling may aid in the optimization of liposome design parameters, such as PK/disposition, drug release rate, and cellular uptake, thus maximizing the delivery of encapsulated drugs at the site of the tumor.

The objective of this work is to articulate a role for mathematical modeling as a tool to: (i) identify and characterize quantitatively the key determinants and processes that dictate the PK and antitumor activity of liposomal formulations; (ii) streamline the development of optimized liposomal formulations; and (iii) aid in the rational development of therapies that combine conventional oncology drugs with nanoparticulate-embodied agents. The principle physicochemical and functional properties of the drug/liposome carrier complex that influence PK and biodisposition most heavily will be explored. Examples will be identified in the literature in which PK/PD models contributed to the understanding of performance-determining characteristics of liposomal anticancer agents and their optimization. Finally, multi-scale systems analysis approaches will be highlighted that seek to bridge quantitatively from preclinical models to human clinical application, and reveal the role of tumor- and drug-carrier system parameters that control tumor drug delivery.

## 2. Requisite Drug and Carrier Properties

The effectiveness of liposomes as oncology drug carriers depends on a balance among numerous factors, such as stability in the circulation, ability to access the target site, and ability to release the drug at the site of action in the tumor. Multiple criteria must be considered in matching properties of the drug with those of the liposome carrier.

First, the drug must demonstrate activity against the chosen tumor type. Common examples include anthracyclines and *Vinca* alkaloids, which have shown activity against a broad range of cancers [14,15,22,23,63].

Second, the loading of the drug in the liposome carrier must be both adequate and, for commercial viability, efficient. Sufficient drug must be incorporated in the carrier to permit delivery of a pharmacologically active dose with an acceptable amount of carrier lipids. The use of active loading techniques increases both cargo capacity and efficiency of encapsulation. An example is the strategy of employing pH and/or electrochemical gradients coupled with a trapping agent within the liposome interior, which can raise encapsulation efficiency for amphipathic basic amines such as DXR or vinblastine to essentially 100%, with high drug:lipid loadings [64,65,66,67,68,69,70].

Third, the drug must be transported stably by the carrier in the circulation yet be released from the carrier at the tumor site with the appropriate rate. The physicochemical properties of the chemotherapeutic agent to be incorporated into the carrier play an important role in determining its release rate.

Candidate drugs can be classified into three categories based on their hydrophobic properties, as reflected in their octanol:water partition coefficient (*K*_p_): (i) highly hydrophilic drugs (*K*_p_ < 1); (ii) highly hydrophobic drugs (*K*_p_ > 3); and (iii) amphipathic drugs of intermediate *K*_p_. Examples of the first category include cytosine arabinoside, cisplatin, methotrexate and *N*-(phosphonacetyl)-l-aspartate. Liposomal formulations designed with these types of drugs may have low capture efficiency if passive encapsulation is employed. Such formulations may be highly stable *in vivo* with the appropriate liposome composition, and release rates may require optimization to provide the appropriate bioavailability at the tumor site. Some strategies to control drug release rates revolve around designing carriers that are stable in plasma but rapidly destabilized in the tumor to facilitate the delivery of free drug.

The second category of drugs tends to intercalate into the liposome bilayer, improving their overall solubility and often their biodistribution, but rendering the drugs prone to rapid extraction or exchange with plasma proteins after administration. These drugs may not be appropriate for active targeting strategies if the release rate is high.

The third category of drugs would also have relatively rapid release rates because their intermediate partition coefficients confer moderate water solubility and membrane permeability. However, in combination with loading methods that entrap drugs stably in the liposome interior, they may be highly suitable for liposome-based delivery. This group of agents includes somewhat lipophilic cationic drugs such as the anthracyclines (DXR, daunorubicin), anthracenediones (mitoxantrone), *Vinca* alkaloids (vincristine, vinblastine, and vinorelbine), camptothecins, and numerous others. With this drug category, it is possible to modulate the drug-release rates by employing gradient-based loading techniques in order to maintain stable encapsulation in the circulation, yet allow the drug to be released at the tumor site [14,65,66,68,71,72,73,74].

## 3. Integration of *in Vivo* Factors Influencing Pharmacokinetic (PK) and Performance of Liposomal Formulations

The interaction of liposomes with *in vivo* systems is enormously complex and involves many factors that affect therapeutic performance. Total systemic clearance is often regarded as the most important global pharmacokinetic property owing to its inverse relationship with net drug exposure and the average steady-state plasma concentration achieved during continuous administration. Net drug exposure is the area under the drug concentration-time curve (AUC). Continuous administration can include both constant-rate of infusion and/or multiple-dosing regimens. The overall clearance of liposomal formulations is dependent upon three factors: (i) the rate of elimination of the liposome carrier itself; (ii) the rate of release of the encapsulated or membrane-incorporated drug from the carrier; and (iii) the rate of elimination and metabolism of released drug that is no longer associated with the carrier.

The processes by which liposomes and other nanoparticles are cleared from the bloodstream have been investigated in considerable detail [75,76]. The main mechanism for their elimination is through recognition and uptake by macrophages of the reticuloendothelial system (RES), which reside primarily within the liver and spleen [77,78,79,80]. Liposome clearance may be influenced by physicochemical factors that are discussed below, including the vesicle size, lipid composition of the membrane, and release rate of liposome contents. Liposomes can also interact with plasma constituents such as proteins, thus affecting their fate *in vivo*, either by affecting their stability and/or modulating their subsequent interaction with the target cells [75]. Plasma protein interactions may extract or exchange lipids from the carrier, compromising its integrity. Mediators of clearance include plasma protein opsonins, fibronectin, C-reactive protein (CRP), the C3b complement fragment, β2-glycoprotein I, or the Fc portion of an immunoglobulin G (IgG) [77,81,82]. Opsonization can compromise liposome stability and promote endocytosis or phagocytosis by macrophages of the RES. Renal excretion may represent a significant component of total clearance for very small nanoparticles (<4–8 nm diameter) but does not impact clearance of most liposome formulations (>30 nm) [83,84].

The stability of drug association with the liposome has the potential to alter substantially the circulation half-life and other pharmacokinetic properties of the drug, depending upon the relative magnitude of differences in the factors controlling drug and liposome disposition. Efforts to promote the internalization of drug-loaded liposomes by cancer cells can raise efficacy (below), but local release of drug from extracellular liposomes can provide the main component of therapeutic efficacy in some applications. For these reasons, drug release from liposomes has major impact upon toxicity and antitumor efficacy [3,85]. A number of studies identify the kinetic complexities arising from the rate of tumor accumulation of liposomes and the rate of drug release, and show that it may be necessary to modulate the rate of drug release for optimal efficacy [71,74,86]. Measurement of *in vivo* drug release rate is enormously important in the development of liposome formulations but can be highly challenging for certain types of drugs. Total plasma concentration usually can be quantified by conventional technologies. Released drug has been measured directly by numerous methods including microdialysis or solid-phase micro-extraction techniques [87,88], both of which are tedious and expensive. Indirect approaches for measuring *in vivo* drug release rates include simply measuring changes in the plasma drug-to-liposome ratio [21,89], which is valid for cases in which the clearance rate of the released drug from plasma is sufficiently faster than the release rate from liposomes. For hydrophobic drugs, or drugs having a high fraction of plasma protein binding, measurement of released drug can be exquisitely challenging, but nonetheless an extraordinarily important component for understanding the parameters affecting dosage form performance. In the absence of measured drug release kinetics, strategies employing modeling of pharmacokinetic profiles for liposomal- and unencapsulated drug may provide indirect *in vivo* estimates of the drug release rates. One recently described indirect approach permitted inference of drug release rates based upon measurements of the free fraction of drug in plasma (*i.e.*, the fraction of released drug unbound by plasma proteins) [90], which was employed successful in a PK modeling application for a nanoemulsion paclitaxel formulation. A second approach employed simple compartmental modeling of liposomal- and unencapsulated amphotericin B PK to estimate a single, species-independent first-order release rate constant for rats and humans [91].

The accumulation of liposomes in tumors is controlled by numerous processes, including tumor perfusion, extravasation into the tumor tissue, and transport within the interstitium [92,93]. The permeability of the tumor microvasculature governs the influx and efflux of drugs [92,94,95,96,97]. Most liposome deposition results from the EPR effect [26], in which the discontinuous, permeable tumor vascular endothelium, and impaired lymphatics surrounding the tumor [26,98,99,100,101], permit extravasation and accumulation. Drug exposure to healthy tissues (and associated toxicities) is limited by the integrity of the normal tissue vasculature to nanoparticulates. Compared to extravasation *via* the discontinuous endothelium of the tumor microvasculature, transcytosis of liposomes through vascular endothelial cells is thought to represent a relatively minor pathway. High tumor interstitial fluid pressure also limits liposome diffusion into tumors.

Once in the tumor, most liposome compositions tend to remain in close proximity to the microvessels from which they extravasated because of the high tumor interstitial pressure, the dense extracellular tissue stromal matrix, a large interstitial space compared with normal tissues, and the low diffusion coefficient of nanoparticulates [93,95,96,97,99,101]. The high interstitial pressure of larger tumors has been reported to reduce liposome distribution into the tumor interstitium in comparison to smaller tumors, and, as expected for regions of tumor into which oxygen and nutrients do not readily penetrate, there is little liposome penetration into necrotic regions of tumors [95].

The intracellular delivery of encapsulated drugs appears to enhance the efficacy of numerous agents. It is generally accepted that the majority of liposomes enter cells through the endocytic pathway, which is supported by both direct and functional evidence [102,103,104,105,106]. However, the molecular mechanisms of cellular binding and internalization of liposomes are not yet fully understood. If targeting cell surface receptors that are endocytosed can enhance intracellular delivery, tumor cell killing and antitumor efficacy can be increased [107,108,109,110]. Intracellular release of drug from the endocytic compartment is a necessity, and various approaches to enhance release to the cytoplasm have been investigated. One involves pH-triggered strategies that exploit the acidic tumor microenvironment or acidic endosomal compartments to destabilize liposomes [111,112,113]. Another example employs the selection of agents that require a liposome carrier for optimal delivery to the endocytic compartment; such “liposome dependent drugs” are polar, weakly acidic drugs that become more membrane permeable at endosomal and lysosomal pH [105,106,114].

## 4. Physicochemical Properties of Liposomal Formulations and Their Effects upon Pharmacokinetics

Numerous physicochemical properties of liposomes influence their pharmacokinetics directly or indirectly, and are discussed below.

### 4.1. Particle Size

In general, the clearance of liposomes and other nano- or micro-particulates from the blood increases with increasing particle size [85,115,116], and clearance is mediated principally by the RES. Smaller liposomes tend to have longer circulation half-lives than larger liposomes of the same membrane composition, and distribute more rapidly in solid tumors due to their enhanced ability to extravasate across the more porous tumor endothelia [85,116]. It has been suggested that despite their slower clearance from the circulation, smaller liposomes (60–80 nm) tend to accumulate in tumors less efficiently than slightly larger liposomes [115,116]. Thus the longer circulating lifetime of the smallest liposomes may be counter-balanced by an increase in reversible permeability (enhanced influx and efflux rate constants), and therefore the role of diameter in optimizing liposome deposition may depend upon an interplay with additional variables. Liposomes of approximately 100 nm diameter have been reported to distribute into solid tumors with an efficiency that is partly dependent on the anatomical location of the tumor [117].

Liposome surface properties modulate the degree to which liposome size influences total blood clearance. Clearance of sterically stabilized liposomes of relatively large diameters (80–250 nm), such as SSL that bear a polyethylene glycol corona on their surface, is less sensitive to the effects of particle size than is clearance of non-pegylated liposomes of equivalent size [118,119]. Naturally occurring glycolipids and glycophospholipids can exert similar effects [120,121]. Maximum accumulation of sterically-stabilized liposomes in xenograft animal models typically occurs between 24 and 48 h post administration [96,108,120].

### 4.2. Membrane Charge

The incorporation of high molar ratios of charged lipids accelerates the clearance of liposomes. Negatively charged liposomes commonly include anionic phospholipids such as phosphatidic acid (PA), phosphatidylserine (PS), and phosphatidylglycerol (PG). Positively charged liposomes are commonly composed of cationic dialkyl/diacyl lipids such as dioleoyl-trimethylammonium-propane (DOTAP), di-octadecenyl-trimethylammonium-propane (DOTMA) and other analogs. Stearylamine has been used historically as a constituent of cationic liposomes, but is more cytotoxic than dialkyl or diacyl lipids, and more likely to undergo extraction from the membrane or inter-membrane exchange [122,123,124].

Negatively charged liposomes tend to be removed from circulation by the macrophages of the RES, and increasing charge increases total systemic clearance [77,125]. A notable exception is the anionic glycerophospholipid phosphatidylinositol (PI), which can mediate a longer circulating lifetime because of the “steric stabilization” effect of its inositol headgroup [96,119,120]. Positively charged liposomes are cleared rapidly by the RES, but also bind rapidly to vascular endothelium, sites of inflammation, and plasma proteins [126,127,128].

### 4.3. Membrane Lipid Composition and Surface Properties

The combination and physicochemical characteristics of lipids from which liposomes are prepared are critical in influencing the pharmacokinetics of the carrier and the associated drug. Liposome physicochemical properties can influence total drug clearance in several ways. The first is by modulating directly the rate of liposome recognition and elimination by cellular clearance mechanisms and tissues of the RES. The second is by modulating the release rate of drug from the carrier, which results in pharmacokinetics that reflect the circulating life-time of the free drug; small-molecule drugs tend to be cleared relatively rapid compared to liposomes that have been optimized for extended circulation times. Both drug release and liposome clearance by the RES are reduced by employing high phase-transition (high T_m_) phospholipids such as distearoyl or hydrogenated phosphatidylcholines (PC), and the inclusion of cholesterol (Chol): decreased binding of plasma opsonins reduces RES clearance through receptor-mediated pathways, and a reduced ability of proteins to insert into cholesterol-rich, high T_m_ membranes reduces drug leakage [77,129,130]. Sphingomyelin (SM) also can exert a stabilizing effect on the phospholipid bilayer, and the combination of SM/Chol has been reported to extend the circulation lifetime of liposome-encapsulated drugs compared to the same drugs in distearoyl-phosphatidylcholine (DSPC)/Chol liposomes [131]. Surface modifications that sterically stabilize liposomes and extend circulating lifetime have been described above.

### 4.4. Operational Categorization of Liposomes

The wide range of liposome characteristics and performance that can be produced by blending these and additional properties is a considerable advantage of this carrier system. Conceptually there may be several operational categories of liposomes based on composition and *in vivo* utilization (Figure 1). This grouping is not exclusive or absolute.

#### 4.4.1. Conventional Liposomes

First-generation liposomes developed as a drug carrier system fall into this category. Composed of charge-neutral and/or negatively charged phospholipids, such liposomes typically have short blood circulation times. After intravenous administration, these liposomes undergo protein binding and insertion, phospholipid exchange, opsonization, rapid uptake by macrophages, and accumulation in tissues of the RES. Rapid clearance and low stability of encapsulation can limit seriously their application for the treatment of many diseases. Exceptions exist; one is that such liposomes may be effective for increasing the apparent solubility of highly lipophilic drugs; a second is where delivery to macrophages or to the RES is an application, for example, where the objective is delivery of immunomodulators to macrophages in order to enhance their capability to kill neoplastic cells [132].

#### 4.4.2. Sterically-Stabilized Liposomes (SSL)

The development of long-circulating liposomes represents a significant advance in liposomal drug carriers [133]. The advantage of SSL is their ability to extravasate into solid tumors due to the EPR effect. As described above, a variety of covalently-linked, hydrated, surface-modifying groups delays clearance by the RES and results in prolonged circulation times (≥24–48 h).

#### 4.4.3. Immunoliposomes

Receptor-specific ligands, typically immunoglobulins or their subunits, have been investigated as a means to promote tumor deposition as well as tumor cell uptake of liposomes. A growing consensus suggests that the magnitude of tumor deposition attributable to the EPR effect may be greater than the contribution of ligand-based arrest within the tumor, and that the major advantage of the targeting ligand is to promote liposome endocytosis and intracellular delivery [108,134,135,136,137,138,139,140]. The necessity for the drug to escape the endocytic pathway suggests an advantage of the enhanced permeability of “liposome-dependent” drugs [105,106,114], many of which are weakly acidic and would be more permeant at acidic pH of endosomes/lysosomes. However, weakly basic drugs such as the anthracyclines and *Vinca* alkaloids have been used quite successfully in endocytosis-prone liposomes, despite the fact that they would be less membrane permeant at acidic pH. Ligand-targeted liposomes, being highly multivalent, show much higher affinities for target cells than do the individual ligands [141]. A recent example demonstrated that the anticancer activity of human epidermal growth factor receptor 2 (HER2)-targeted immunoliposomes is relatively independent of the intrinsic affinity of the receptor for its cognate ligand: despite relatively low binding affinity of the targeting ligand (*k*_D_ = 160 nM), the targeted liposome construct showed improved efficacy as a result of its efficient internalization [137].

#### 4.4.4. Cationic Liposomes

The initial development of these liposomes was focused on delivery of complexed nucleic acids to the cell interior, thus promoting delivery and expression of these large, highly hydrophilic, negatively-charged molecules. Because of the propensity of cationic liposomes to bind to vascular endothelium and sites of inflammation (above) they may have additional applications in oncology drug delivery.

## 5. PK/ Pharmacodynamic (PD) Analysis of Liposomal Formulations

Unmodified first-generation liposomes typically exhibit nonlinear, saturable PK after intravenous administration, with relatively short elimination half-lives at low, non-saturating doses [142]. Although sterically-stabilized liposomes may show linear PK over a wide range of doses [142], their PK and biodistributional pattern is also complicated at low doses and upon repeated administration schedules [143,144,145,146,147,148]. It is hypothesized that at lower doses, the differences in plasma half-lives between conventional liposomes and SSL should result in a greater pool of SSL available for tumor deposition. For higher doses, the saturable elimination processes for conventional liposomes should be at-capacity, and therefore differences in clearance and tumor deposition among various liposome types should be reduced. The factors modulating the PK behavior of drugs in conventional liposomes and SSL include physicochemical properties of the liposomal carrier as discussed above, the dose, dosing schedule, the route of administration, the specific drug that is encapsulated, as well as the presence of targeting ligands or other surface-displayed molecules, all of which can affect the PK of the liposomal carrier or retention of the drug.

### 5.1. Mathematical Modeling of Liposomal Anticancer Drugs

The PK of SSL formulations of DXR are clearly distinct from that of unencapsulated DXR [149]. The SSL formulation has an extended circulating lifetime and a pattern of tumor- and tissue distribution that reduces drug deposition in heart, a major organ of DXR toxicity; it also increases deposition in skin [115], which appears to correlate with a novel adverse effect of SSL-DXR [150,151].

Arnold et al developed a simple PK model for brain tumor deposition of SSL-DXR in rats [152], which consisted of a one-compartment model having linear elimination from the plasma that was linked to a peripheral compartment representing the site of action (*i.e.*, the brain tumor). The context for development of the model was to understand why specifically-timed dosing schemes with SSL-DXR mediated a progressive increase in brain tumor vascular permeability, and resulted in elevated deposition of subsequent SSL-DXR doses [144]. The objective was to identify quantitatively the pharmacokinetic factors that might underlie the elevated deposition of subsequent doses in this “tumor priming” administration schedule. Therefore, the model included explicit tumor influx- and efflux rate constants for unencapsulated DXR and SSL-DXR. The final model reasonably captured plasma and brain tumor concentration-time profiles of DXR for both formulations, and for single- and multiple-dosing schemes. The analysis supported the conclusion that in rats administered a dose of SSL-DXR one week previously, intratumor DXR concentrations increased more rapidly, and to a greater extent, compared to naïve (previously-untreated) animals. The model estimated that the prior SSL-DXR treatment elevated tumor vascular permeability and increased the SSL-DXR influx rate 4-fold, whereas the efflux rate increased by 2-fold. The net result was an overall increase in tumor drug deposition that agreed well with experimental data. The model also captured an observed shift in the peak time of SSL-DXR accumulation to earlier time points, consistent with elevated tumor vascular permeability. Although deposition in the brain tumor as well as elimination processes were relatively well described, the model did not distinguish between the delivery of liposome-encapsulated drug and liposome-released drug. Nor did it account for the release kinetics of DXR from the liposome.

**Figure 2:**
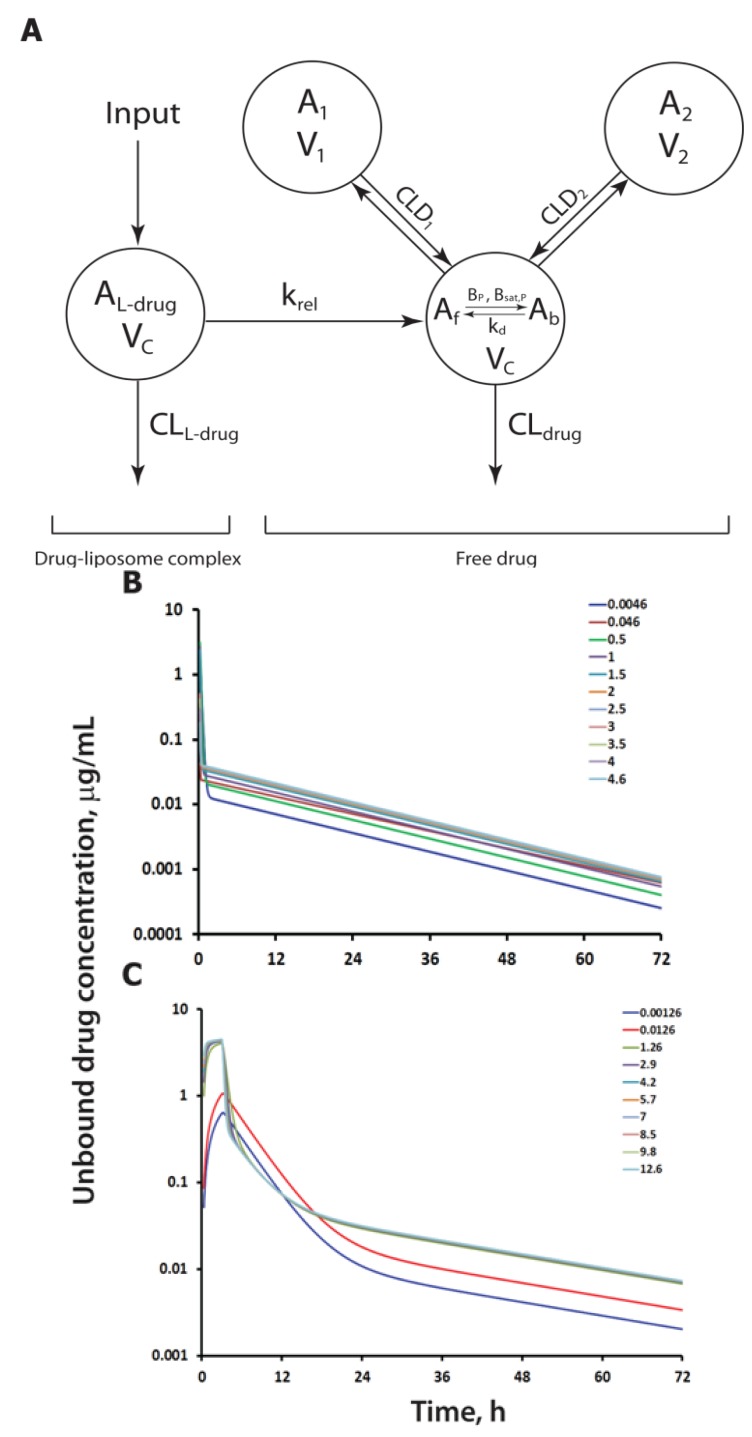
General pharmacokinetic (PK) model diagram for liposomal drug formulations and model simulations of released drug concentration as a function of time. (**A**) The PK of LEP-ETU (liposomal paclitaxel) was captured using a three-compartment model, whereas a two-compartment model sufficed to describe the PK of sterically stabilized liposomes (SSL)-DXR (liposomal doxorubicin). In the model, the drug-containing liposome (L-drug) is administered into the blood (*R*_input_); it circulates within the compartment *A*_L-drug_ and can undergo clearance with drug still encapsulated (CL_L-drug_). The liposome releases drug according to a first-order release rate constant (*k*_rel_). Drug equilibrates between protein-bound (*A*_b_) and unbound (free) (*A*_f_) states, and distributes to peripheral tissues (compartments *A*_1_, *A*_2_) with inter-compartment clearances from the first peripheral compartment (CLD_1_) and from the second peripheral compartment (CLD_2_). For both liposomal drug formulations, the released drug is eliminated from the central compartment with a linear clearance CL_drug_. For LEP-ETU, paclitaxel binds to plasma proteins in both a linear (*B*_p_) and saturable (*B*_sat_) manner, depending upon concentration, whereas only linear binding is required to describe DXR; (**B**) Model simulations for a 10 mg/kg dose of SSL-DXR administered i.v. to mice, showing the change in plasma concentrations of unbound DXR as *k*_rel_ parameter is varied over a 1000-fold range around the value for SSL-DXR (0.046 h^−1^) obtained from the analysis of [153]; and (**C**) Model simulations for a 175 mg/m^2^ dose of LEP-ETU administered intravenously (i.v). to humans by infusion over a 3 h period, showing the change in plasma concentrations of released paclitaxel (PAC) as *k*_rel_ is varied around the value for LEP-ETU (1.26 h^−1^) obtained from the analysis of [159].

A subsequently developed PK model for SSL-DXR (Figure 2) [153] included estimates of drug release rate along with tumor drug delivery. It was developed in the context of understanding PK/PD relationships for SSL-DXR in a “tumor priming” combination strategy, in which a prior dose of paclitaxel increased tumor permeability, as well as deposition and intra-tumor diffusion of SSL-DXR [154]. Data for tumor deposition that discriminated liposome-encapsulated *vs.* released DXR were not available. Therefore, the model assumed, as a simplification, that the release of DXR from liposomes to the plasma is governed by a linear first-order process. Once in the plasma, free drug could undergo linear clearance, distribute into a peripheral systemic compartment, or partition into a tumor compartment. In the tumor compartment, SSL-DXR concentrations were defined by a well-stirred model. The final model not only captured the data well, but also estimated the half-life of DXR release from liposomes to be approximately 15 h (first order release constant *k*_rel_ = 0.046), which is consistent with other studies of *in vitro* and *in vivo* drug release rates for similar liposome formulations [71,155].

Another approach to characterizing PK of free and liposomal SSL-DXR is a so-called “hybrid model”, in which plasma disposition is described by traditional compartmental models, but plasma PK is linked to tumor drug concentrations using physiologically-based pharmacokinetic (PBPK) models that include mechanism-based terms such as tumor blood flow and tissue volume [156,157]. Model sensitivity analysis clearly identified the release rate of DXR as an important parameter for the optimization of liposomal delivery. Interestingly, after scaling the PK model from rodents to humans, preliminary simulations suggested species differences in the optimal rate of drug release. The concept of species-dependent differences in SSL-DXR PK is developed further in sections that follow.

Finer details of drug disposition have been considered in other modeling approaches. An intracellular PK model for DXR was applied to a SSL-DXR formulation [158]. Briefly, the model assumed that a mass transfer coefficient, the cell surface area, and a partition coefficient together determine DXR penetration into the cell membrane. Assumptions were that transport from the outer leaflet to the cytosol was mediated through flippases, and cytosolic DXR reversibly binds to DNA with roughly 0.18 binding sites per nucleotide. The intracellular model for DXR transport was implemented within the cellular compartment of the tumor model. Furthermore, it was possible to scale the full PK model, which included plasma, tumor, and cancer cell components, to humans by adjusting the PK parameters for free- and liposomal DXR to values derived from clinical data, as discussed below.

### 5.2. Effect of Drug Release Rate on Plasma PK

Drug release rates have enormous impact on biodistribution of liposome-associated drugs and on the performance of liposome-based formulations. Release rates can vary with the composition and type of liposome, the method of drug incorporation or encapsulation, and the specific properties of the drug itself. Highly hydrophobic drugs, which are generally incorporated into the liposome membrane rather than entrapped in the interior aqueous core, typically have high release rates. They can partition reversibly among plasma (or interstitial fluid), plasma proteins, and liposomes, and can be stripped by liposome interactions with hydrophobic “sinks” such as serum proteins, lipoproteins, cells in the plasma, or cells in tissues. Few robust, broadly-applicable analytical approaches have been developed to measure the release of hydrophobic, membrane-incorporated drugs from liposomes, despite the fact that drug release rates determine the biodisposition of the drug, and if sufficiently rapid, can render carrier-targeting approaches useless for controlling target deposition of drug.

To investigate the impact of drug release rates on the plasma pharmacokinetics of a liposome-associated drug, model simulations were generated for the time-course of unbound drug concentrations for two very different liposomal formulations (Figure 2): (i) LEP-ETU, a liposome-based formulation of paclitaxel (PAC), in which the drug is intercalated in the membrane bilayer; and (ii) SSL-DXR, a highly stable formulation in which doxorubicin is encapsulated in the aqueous interior by a “remote loading” pH/electrochemical gradient procedure [67,160,161]. Unbound drug was selected for estimation because the fraction of released drug that is not bound to plasma proteins is the pharmacologically active form. Figure 2A shows a general schematic of the two models that were utilized [153,159]. The model assumptions are that liposomal drugs (L-drug) are injected into the vascular compartment while incorporated within a liposomal carrier “compartment” (*A*_L-drug_), from which free drug (*A*_drug_) is released through a first-order process having a rate constant (*k*_rel_). Encapsulated drug that remains within the liposome can also be cleared with a linear clearance (CL_L-drug_). Carrier-released drug can exist in a free, unbound state, or can bind to plasma proteins in either a linear fashion, with a constant plasma-bound fraction (*B*_p_) (as assumed for DXR), or with both linear- (*B*_p_) and capacity-limited saturable bound fractions (*B*_sat_). An equilibrium dissociation constant (*K*_D_) accounts for those cases in which drug equilibrates between the unbound (free) and bound states, as assumed for PAC. Free drug is cleared from the blood linearly with a clearance rate (CL_drug_), or distributes to one or more peripheral compartments (*A*_i_) via a distributional clearance rate (CL_Di_).

In the first set of simulations, the mean estimated drug release term *k*_rel_ for SSL-DXR was varied 1000-fold around a baseline value for *k*_rel_ of 0.046 h^−1^ (release half-life of 15 h), which was determined from a previous analysis of SSL-DXR PK data in various model systems [153]. The simulations showed that increases in *k*_rel_ up to 100-fold above the baseline value had relatively little effect upon unbound DXR concentration or the duration of the distribution phase. In contrast, decreasing *k*_rel_ 10-fold below the baseline value (to 0.0046 h^−1^) resulted in a decrease in unbound DXR plasma concentrations and a progressive lengthening of the distribution phase (Figure 2B). These simulations demonstrate that the distribution of unbound drug is better controlled with a slow drug release rate.

A second set of simulations examined the effect of changes in *k*_rel_ for paclitaxel in LEP-ETU. A previous analysis of published clinical data for four paclitaxel formulations estimated *k*_rel_ values in the range of 0.7 to 5.2 h^−1^; a baseline *k*_rel_ of 1.26 h^−1^ (0.55 h half-life) was estimated for PAC in LEP-ETU [159]. Simulations with the model (Figure 2C) show that increases in *k*_rel_ up to 10-fold above the baseline have little effect on the PK profile of unbound PAC. In contrast, decreasing *k*_rel_ to values 100-fold below the baseline (from 1.26 h^−1^ to 0.0126 h^−1^) results in progressive decreases in unbound plasma drug concentrations, and a prolongation of the distribution phase.

A hypothesis based upon these simulations is that the mean estimates of *k*_rel_ for SSL-DXR [153] and LEP-ETU [159] are close not only to their optima, but also to threshold values beyond which further changes in *k*_rel_ alone are unlikely to result in an improved therapeutic outcome without concomitant changes in other formulation characteristics. Additional ramifications are discussed below.

### 5.3. Effect of Drug Release Rate on Tumor PK

The mechanisms by which liposomes release drug in tumors have not been elucidated well. Several hypotheses have been proposed, but little direct evidence has been provided, primarily because of the difficulties of monitoring drug release within tissues *in vivo*. Several factors may influence liposome destabilization and drug release, including the tumor microenvironment, the pH of the interstitial fluid surrounding tumor parenchyma (typically acidic compared to plasma pH), enzymes released from apoptotic or inflammatory cells (such as lipases), and oxidizing radicals that may be released during an immune response against the tumor [21]. *In vitro* studies suggest that cellular transporters can extrude cytoplasmically-released compounds from the cell or that endocytosed drug may be regurgitated during endocytic vesicle recycling [162,163]. *In vivo*, macrophages residing within the tumor can degrade liposomes and liberate the encapsulated drug [4]. One study investigated the effect of interstitial fluid on the release of DXR from SSL-DXR *in vitro* by harvesting malignant pleural effusions [149]. The rate of drug leakage appeared to be faster in malignant effusions than in plasma (*t*_1/2_ = 1 *vs.* 100 h). That work also described a method for selective destabilization of liposomes within the tumor environment as a promising approach to control and increase the delivery of free drug at the tumor site, thereby improve efficacy.

### 5.4. Effect of Drug Release Rate on Antitumor Efficacy

Despite strong correlations between circulation half-life and antitumor activity of liposomal drugs, the relationship between *in vivo* drug release rate of liposomes and antitumor activity is more complex. In a comprehensive study evaluating several liposomal DXR formulations, increasing liposome stability was associated with increased total drug and released free drug in the tumor, and increased therapeutic efficacy [71,73,85,164]. Similarly, the antitumor pharmacodynamic effects of two liposomal formulations of irinotecan, having slow- and moderate release rates (*t*_1/2_ of release = 58.6 h *vs.* 14 h), were evaluated in mice bearing human colon carcinoma xenografts. The slow release formulation, which used sucrose octasulfate (SOS) as an intra-liposomal drug-trapping agent, was more efficacious than the moderate-release formulation, which used polyphosphate to trap the drug instead. Published data also suggest a higher-stability vincristine formulation in sphingomyelin/cholesterol liposomes was more efficacious than a less stable formulation of DSPC/cholesterol liposomes [89].

Slow- and moderate-release formulations (*t*_1/2_ of release = 27.2 h *vs.* 15 h) of an anti-HER2 targeted immunoliposome of vinorelbine showed analogous behavior in HER2-overexpressing breast cancer (BT474) xenografts, with reduced efficacy for the faster-release form. For immunoliposomes, the stability of the phospholipid bilayer is more important than for other types of liposomes, because the drug must remain entrapped until targeting is achieved. Immunoliposomes having more rapid drug release rates could demonstrate antitumor activity if they are targeted to antigens that are easily accessible from the vasculature, such as may be the case for leukemias and lymphomas, or to micrometastases in which extravasation is rapid [165,166,167]. However, DXR-containing immunoliposome formulations bearing anti-CD19 and having varying drug release rates (*t*_1/2_ of 1.9 h to 315 h) were compared in a B-cell lymphoma model. The antitumor activity correlated inversely with the drug release rates [168]: immunoliposomes with the most rapid DXR release rate demonstrated little efficacy, whereas the most stable formulations showed the greatest activity.

Where they have been established experimentally, correlations between *in vivo* liposome drug release rates and antitumor efficacy may not translate across animal species. First, liposomal drugs generally show longer plasma circulation half-lives in human than in rodents (e.g., SSL-DXR has a circulation *t*_1/2_ of 56–59 h in humans [150,169] *vs.* 21–23 h in rodents [169]), suggesting that greater stability may be required in humans in order to maximize tumor deposition. Second, the tumor growth rate generally is slower in humans than in most animal xenograft models. Thus a therapeutically optimal release rate may be considerably faster in mice, in which efficacy may be driven by how rapidly drug is delivered to the tumor and establishes its pharmacological effect. Rapid tumor growth may favor more rapidly-releasing formulations, as has been concluded in analysis such as [156], which is discussed in greater detail below. In slower-growing human tumors, sustained delivery or extended exposure of the tumor to released drug may be more efficacious than rapid, short-term exposures. This situation may be analogous to preclinical experiments that consistently demonstrate improved antitumor efficacy of long-circulating, slow-release SSL-DXR in mouse tumor models compared to equivalent doses of free DXR; free DXR rapidly establishes high peak levels in tumor but clears quickly from the tumor when blood concentrations of drug decline [170]. Interestingly, a recent multi-scale analysis suggest that in humans, free DXR may be more efficacious than SSL-DXR under certain specific conditions relating to tumor perfusion and vascular permeability [110]. Overall, there are few studies that permit direct and quantitative comparison of the effect of *in vivo* drug-release rates for liposomal formulations upon the observed efficacy in preclinical species *vs.* humans. Further studies, supported by mathematical PK/PD and systems pharmacological modeling, are required to establish the conditions under which animal studies that optimize liposomal drug release rates for antitumor efficacy correlate with human clinical outcomes.

### 5.5. Influence of Liposomal Drug Deposition on Antitumor Efficacy

The influence of tumor drug deposition and the effect of liposomal lipid composition on therapeutic efficacy was evaluated for DXR using human tumor xenografts in immunodeficient mice [171]. A pegylated SSL-DXR formulation was shown to be more effective than free DXR and other non-pegylated liposomal formulations of DXR, and SSL-DXR efficacy was observed at doses lower than the maximal tolerated dose of free DXR, indicating a net therapeutic advantage for the liposomal formulation. A subsequent study compared the activity of escalating doses of SSL-DXR *vs.* free DXR in a xenograft mouse model of Lewis lung carcinoma. SSL-DXR at 1–2 mg/kg was equipotent in activity to free DXR at 6 mg/kg, indicating a 3–6-fold increase in drug efficacy [172]. Similarly, the 50% lethal dose of SSL-DXR was nearly twice that of free DXR after a single intravenous injection in mice [68], and in rabbits, the cardiac toxicity of SSL-DXR was considerably less than that of free DXR in a multiple dose study [173].

Liposome-mediated drug delivery has limitations, given that drug transport and deposition are controlled by tumor structure and drug-specific properties [174,175]. Whereas drug transport *via* the blood stream and across the tumor vascular barrier is dominated by convection, a transport mechanism that is relatively insensitive to molecular mass, the subsequent distribution into the tumor interstitium after extravasation is dominated by diffusion, which is slower than convection and heavily influenced by molecular mass or radius of hydration [174]. With relatively few exceptions, there exists a general lack of quantitative, model-based analysis of the dynamic interplay of liposome deposition and delivery events within the tumor. Several compartmental and physiologically-based models have been used to quantify transport processes across rodent and human scales [176], and mechanism-based models have been developed that integrate drug interactions with cell-specific surface receptors and ligands [177]. 

One investigation developed a compartmental model to analyze and compare the performance of unencapsulated DXR, and DXR encapsulated in SSL *vs.* thermo-sensitive liposomes, at different doses, and with bolus injection *vs.* continuous infusions of varying duration [178]. Relationships were developed between plasma and tumor concentrations of drug, and rates of drug equilibration into tumor cells were estimated. Simulations with the model suggested that continuous infusion of DXR is superior. The antitumor efficacy of SSL-DXR was estimated to approach the efficacy of continuous-infusion DXR only if drug release rates are optimal. However, the optimal release rate estimated is more rapid than achieved by DXR liposome formulations currently used clinically. Surprisingly, the analysis suggested that the extended circulation time of sterically-stabilized liposomes conferred little therapeutic advantage over conventional liposomes. Model predictions for thermo-sensitive liposomes suggested a potential advantage at some doses, provided the hyperthermia is applied locally and drug leakage in plasma is not accelerated. 

Another investigation developed a hybrid PK model for unencapsulated DXR and DXR encapsulated in liposomes of varying circulation time and drug release rates. The overall biodistributional PK was described by a simple compartmental model that estimated liposome clearance from the blood by leakage and by RES-mediated clearance. The biodistributional PK was linked to a PBPK model that represented drug concentrations in the tumor capillaries, interstitial space, and tumor cells. The model captured the time course of free DXR concentration in the extracellular space, and was linked to a pharmacodynamic model for tumor cell kill kinetics [156]. The simulations suggested that for rats, reducing the rates of RES clearance and drug release enhanced tumor drug delivery and efficacy of liposomes. However, whereas efficacy continued to increase as RES clearance was decreased, there existed an optimum for the drug release rate of approximately 0.06 h^−1^, which matches the release rate of DXR from long-circulating liposomes in rodents. In the model, the optimal drug release rate in rats was independent of the tumor proliferation rate, the sensitivity of tumor cells to DXR, or the tumor blood flow-rate. Interestingly, an exploratory extension of the model to humans showed that in humans, the optimal drug release rate did vary with tumor cell proliferation rate and sensitivity to DXR. For rapidly-dividing tumor cells, a release rate of 0.06 h^−1^ provided better efficacy, whereas for slowly-dividing tumor cells, lower release rates improved efficacy. Tumors that were relatively high in sensitivity to DXR were relatively less sensitive to drug release rate.

A subsequent mechanistic model [158] was developed to analyze tumor delivery of SSL-DXR and unencapsulated DXR that integrated multiple scales and processes, including plasma PK, tumor physiology, subcellular drug transport, and DXR distribution into the DNA of cancer cells. The kinetics of drug biodistribution were described using a one-compartment PK model for liposomes, and a two-compartment model for unencapsulated or liposome-released DXR. It was assumed that liposomal drug was released according to a first-order process. The plasma PK model was linked to a physiologically-based tumor compartment using estimates of tumor blood volumes and flow. The model assumed that for liposomal DXR to be active in tumor cells, the drug must first be released from liposomes, and that after release, it behaved identically to unencapsulated DXR. The cellular transport component of the model assumed that once DXR is released in the tumor, it partitions into the cell membrane and then is reversibly transported from the outer- to inner- leaflet of the membrane via flippases, dissociates into the cytosol, and then binds reversibly to DNA in the nucleus. This cellular model was embedded within the overall mechanism-based PK model. The analysis and modeling demonstrated that liposome PK and deposition can vary based on liposomes and tumor properties, and plays a major role in controlling overall tumor cell exposure to the drug. As discussed further below, a major factor in determining efficacy was the degree to which the EPR effect promoted tumor delivery of SSL-DXR.

### 5.6. Interrelationships of Release Rates, Deposition, and Efficacy

The effects of drug release and tumor deposition are presented separately above, but are highly inter-related in terms of overall efficacy, and have conceptual importance for the design of formulations and therapies of optimal performance. A decrease in *k*_rel_ translates into a slower release of drug from the liposome, and the overall pharmacokinetics of the system will trend toward the pharmacokinetics of the liposome carrier. Conversely, progressive increases in *k*_rel_ result in overall PK trending toward that of the free drug. But the ramifications of these changes in *k*_rel_ are intertwined with the pharmacokinetics of the liposome carrier, and with the potency of the drug relative to the desired effect endpoint. Free, unbound drug is the mediator of pharmacological activity. Therefore, antitumor efficacy and drug toxicity may be altered considerably as unbound drug concentrations, which are driven by *k*_rel_, change. Reducing drug release rates below some threshold value, at which the drug is cleared faster than it is released, means that unbound drug concentrations will decrease. For stable, long-circulating formulations in which tumor delivery of drug-loaded liposomes, rather than tumor exposure to released drug, controls efficacy, reducing *k*_rel_ could lower systemic toxicity. Conversely, for cases in which longer duration of release and sustained exposure to unbound concentrations of a high-potency drug is therapeutically advantageous, reducing *k*_rel_ could reduce efficacy. Reductions in *k*_rel_ could have a second ramification: if release rates are lowered to values at which the clearance rate of drug from the tumor exceeds the release rate within the tumor, then tumor cell delivery (via endocytosis or other mechanisms) will become increasingly important for efficacy.

For shorter circulating liposome formulations, the ramifications of changes in *k*_rel_ are somewhat different. Taxane formulations such as LEP-ETU provide an excellent example of the complexities. The model for LEP-ETU (Figure 2A) includes estimates for clearance of drug-loaded liposomes (CL_L-drug_). The low-T_m_, slightly charged, somewhat large liposomes of LEP-ETU have a short circulating lifetime compared to SSL. As a result, CL_L-drug_ (clearance of drug-loaded liposomes) is significant. Also, *k*_rel_ is relatively high. Therefore, reductions in *k*_rel_ below a certain threshold result in a reduction in unbound plasma PAC concentrations because drug-loaded liposomes, the source of unbound PAC, would be cleared from the blood before they release drug to the plasma. Interestingly, paclitaxel-mediated neutropenia, one biomarker of toxicity, is related to the time-over-threshold-concentration of unbound drug [179]. It appears from the modeling that the unique combination of CL_L-drug_ (liposome PK) and *k*_rel_ for LEP-ETU results in high initial unbound drug concentrations that fall more rapidly below the toxicity threshold than they do with Taxol^®^, the conventional clinical formulation of PAC, and this complex interplay of CL_L-drug_ and *k*_rel_ could be responsible for the lower toxicity of LEP-ETU compared to Taxol^®^ [159]. This is an important hypothesis to test experimentally.

### 5.7. Analysis of Tumor Priming that Promotes Liposome Deposition

One therapeutic strategy to enhance nanoparticulate drug deposition and distribution within solid tumors is “tumor priming”. The concept is that treatment with an initial agent will compromise the tumor:blood delivery barrier so as to permit greater tumor deposition and intra-tumor distribution of a subsequently-administered agent, in this case, nanoparticle drug carriers. Intrinsic to this strategy is that a temporal “window of opportunity” is created that governs the success of delivery of the second agent, and that identifying the optimal inter-dose interval is essential. There are numerous examples of priming strategies that employ chemotherapy drugs, enzymes that degrade tumor interstitial components, inhibitors of specific signaling pathways that reduce tumor stromal density, and others. One recent strategy for which a linked pharmacokinetic and pharmacodynamic analysis was developed employed an initial treatment with paclitaxel, which creates a wave of apoptosis within solid tumors, resulting in altered tumor vascular perfusion/permeability, reduced tumor interstitial pressure and cellular density, and increased intra-tumor diffusion [180,181]. The subsequently-delivered agent was SSL-DXR [181]. It was observed experimentally that a 48 h inter-dose interval increased SSL-DXR tumor deposition, efficacy, and time to tumor regrowth. The reverse, “non-priming” sequence did not show the equivalent efficacy.

In order to develop a hypothesis describing the linkage between PK and PD in tumor priming strategies, a quantitative system pharmacological model was developed using available data from the literature [155,181,182,183,184,185]. The final model [153] captured the observed data [181] for the PK of paclitaxel and SSL-DXR as single agents, as well as effects on tumor drug exposure, cellular responses such as apoptosis, and tumor volume progression when the drugs were administered alone, combined in a priming-inducing sequence, or administered in the reverse, non-priming sequence. One simplification of the model was that for both drugs, apoptosis was assumed to be the sole cause of death, and therefore the time course of apoptosis, which exhibits a considerable lag period, drove pharmacodynamic interactions. An important characteristic of the final model was that in order to capture the PK and PD effects of PAC tumor priming, it was necessary to hypothesize a feedback loop through which the tumor apoptotic response elicited by PAC drove an increase in tumor permeability to SSL-DXR, which resulted in enhanced deposition. For the reverse, non-priming sequence, no feedback loop was required to link initial SSL-DXR deposition to subsequent paclitaxel deposition or effect.

**Figure 3:**
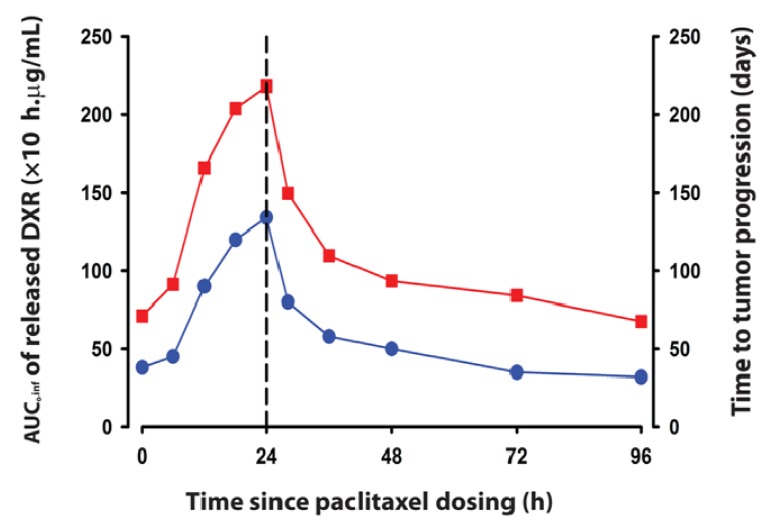
Effect of inter-dose interval between PAC administered for tumor priming upon tumor exposure and efficacy of a subsequently-administered dose of SSL-DXR. Each symbol represents the area under the effect curve (AUC_0-inf_) of DXR released from liposomes (red squares, left ordinate) and the time to-tumor progression (defined as the time for tumor volume to double; blue circles, right ordinate) achieved as the inter-dose interval is varied according to the time indicated by the abscissa. Results are extracted from the analysis of [153].

The PK and PD predictions of the model were highly sensitive to the inter-dose interval, which, in turn, determine the interrelationship between the temporal lag of the apoptotic cascade, progressing from initiation to cell death over a period of 24–48 h, and the changes in tumor permeability that increased deposition and efficacy of the subsequent SSL-DXR dose. Simulations with the final model (Figure 3) suggested that a shorter inter-dose interval than tested experimentally could be optimal for DXR deposition and would increase efficacy drastically, with 2.5-fold greater SSL-DXR deposition in the tumor achieved by halving the 48 h inter-dose interval to 24 h. The model also predicted that efficacy (time to tumor progression) would mirror drug deposition, and would be greatest with a 24 h inter-dose interval. Experimental testing of the model predictions will be valuable. No experimental data existed for other important tumor responses to priming, such as the degree of tumor interstitial pressure reduction and its time course. Reduction in the outward convective fluid force could have major impact on SSL-DXR deposition in tumor priming sequences. This and additional phenomena that are perturbed in tumor priming strategies could be integrated in a future predictive model, and tested experimentally.

## 6. Translation of PK System Parameters from Animal Models to Humans

Multi-scale predictive modeling of parameters controlling liposome formulation performance is not well developed but would be very valuable for bridging preclinical and clinical development of oncology drugs. In some cases, relatively simple approaches can be applied to existing data to obtain species-bridging- or species-independent parameters. One example is a study that investigated a liposomal formulation of the hydrophobic anti-fungal drug amphotericin B. Drug release kinetics were estimated using compartmental modeling that compared the PK of liposomal- and unencapsulated drug formulations. A key assumption was that the micellar deoxycholate drug carrier used for the unencapsulated drug was diluted rapidly to a concentration at which it would no longer influence the PK of the unencapsulated drug, and therefore the PK of the unencapsulated drug represents the PK of drug released from the liposome. A single, species-independent first-order release rate constant (*t*_1/2_ of release = 198 h) was estimated for rats and humans [91]. Drug release kinetics were then incorporated into vascular- and tissue sub-compartments of PBPK models to describe amphotericin B biodistribution in mice and rats, systems for which such data were available, and then to predict the pharmacokinetics of the drug in human tissues.

Alternatively, mechanism-based PK models offer the advantage of identifying drug- and system-specific factors that determine the magnitude and time-course of pharmacological processes. They offer a unique opportunity to integrate simultaneously information obtained from *in vitro* analytical assays and *in vivo* animal studies, and provide a potential means to anticipate both therapeutic and adverse responses to drugs in humans. A multi-scale PK model was developed recently for free DXR and SSL-DXR [158] in mice and humans that is based on mechanistic hypotheses underlying drug delivery for both formulations on organismal, tissue, and cellular levels. The model developed made it feasible to scale and compare PK system parameters obtained from *in vitro* data, preclinical models, and humans, so as to make predictions as to what factors would favor un-encapsulated DXR *vs.* SSL-DXR in humans. Table 2 summarizes the key PK system parameters from [158] that could be scaled to bridge formulation PK between mice and humans. The analysis estimated that SSL-DXR plasma half-lives were approxiamtely 3-fold greater in humans than in mice, whereas the clearance of unencapsulated DXR in humans was approxiamtely 4-fold lower than in mice. Free DXR transport coefficients in mice were estimated based upon human data: clinical studies that employed dynamic contrast-enhanced magnetic resonance (DCE-MR) imaging with gadolinium-based contrast agents allowed the fitting of a coefficient for DXR transport into human tumors (transvascular flux into the tumor for DXR; tvf_in_dox) based upon an underlying assumption that gadolinium has similar diffusive transport characteristics as free DXR [186,187,188]. The coefficient parameter for DXR transport out of the tumor (tvf_out_dox) was also fitted from human data for SSL-DXR and unencapsulated DXR [29]. For DXR, the tumor influx parameter tvf_in_dox was approxiamtely 12-fold greater in humans than mice, and the efflux (tvf_out_dox) was approxiamtely 7-fold greater in humans. For SSL-DXR, the inward transvascular flux coefficient for liposomes (tvf_in_lipo) was estimated from available clinical data [47,189], and the outward transvascular flux (from the tumor interstitial space to the capillary space, tvf_out_lipo) was based upon data from mouse models [115]. After normalization of the data per unit surface area, both inward and outward flux of liposomes was assumed to be equivalent for humans and mice.

**Table 2:** Systems parameters that can be scaled up from animals to humans for liposomal doxorubicin (DXR). Adapted with permission from [158]. Copyright 2014 Nature.

Parameter (unit)	Definition	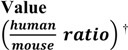	References
*Mouse DXR*			
k12_DXR (1/min)	Rate constant of DXR transport from central to peripheral compartment	0.74 (0.1)	[158]
k21_DXR (1/min)	Rate constant of DXR transport from peripheral to central compartment	5.5 × 10^−3^ (0.2)	[158]
kel_DXR (1/min)	Rate constant of DXR elimination from central compartment	0.36 (0.2)	[158]
tvf_in_DXR (cm/min)	Transvascular flux per surface area for DXR from capillary to interstitial space	2.96 × 10^−4^ (12.3)	[158]
tvf_out_DXR (cm/min)	Transvascular flux per surface area for DXR from interstitial to capillary space	1.18 × 10−3 (7.2)	[158]
*Mouse Liposome*			
kel_lipo (1/min)	Rate constant of liposome elimination from central compartment	1.14 × 10^−3^ (0.1)	[158]
tvf_in_lipo (cm/min)	Transvascular flux per surface area for liposome from capillary to interstitial space	2.64 × 10^−6^ (1.0)	[158]
tvf_out_lipo (cm/min)	Transvascular flux per surface area for liposome from interstitial to capillary space	7.14 × 10^−6^ (1.0)	[158]
*Human DXR*			
k12_DXR (1/min)	Rate constant of DXR transport from central to peripheral compartment	4.75 × 10^−2^ (0.1)	[158]
k21_DXR (1/min)	Rate constant of DXR transport from peripheral to central compartment	1.25 × 10^−3^ (0.2)	[158]
kel_DXR (1/min)	Rate constant of DXR elimination from central compartment	8.2 × 10^−2^ (0.2)	[158]
tvf_in_DXR (cm/min)	Rate constant of DXR elimination from central compartment	3.63 × 10^−3^ (12.3)	[186,187,188]
tvf_out_DXR (cm/min)	Rate constant of liposome elimination from central compartment	8.45 × 10^−3^ (7.2)	[158]
*Human Liposome*			
kel_lipo (1/min)	Rate constant of liposome elimination from central compartment	1.67 × 10^−4^ (0.1)	[47]
tvf_in_lipo (cm/min)	Transvascular flux per surface area for liposome from capillary to interstitial space	2.64 × 10^−6^ (1.0)	[158]
tvf_out_lipo (cm/min)	Transvascular flux per surface area for liposome from interstitial to capillary space	7.14 × 10^−6^ (1.0)	[158]
*Tumor*			
Qtumor (L/min/kg)	Blood flow into tumor	2.82 × 10^−2^ (0.1)	[176,190]

^†^ Fold difference between human and mouse parameter estimates.

By comparing the estimates of liposome deposition parameters for various types of tumors, it was concluded from the system analysis that in some solid tumors such as breast cancers, liposome permeability was lower than in other tumors such as Kaposi’s sarcoma. The overall conclusion from the modeling and analysis was that with certain combinations of characteristics, such as low tumor vascular permeability to liposomes, unencapsulated DXR might be more efficacious than SSL-DXR. The model made clear predictions as to which factors determine superior or inferior therapeutic performance of a liposomal formulation *vs.* unencapsulated drug, and identified parameters that would be important in extending this type of analysis to other liposomal formulations. A data- and mechanism-based analysis for comparing other nanoparticulate formulations with unencapsulated drugs could both speed clinical development and enable stratification of patients so as to treat each with the most efficacious DXR dosage form.

## 7. Conclusions

Significant advances have been made over the past several decades in understanding the physicochemical factors that affect the pharmacokinetics of oncology drugs in liposome-based formulations. Yet development of a quantitative formalism for describing drug- and carrier pharmacokinetic behavior lags in comparison to available literature on other comparatively new therapeutic entities, such as immunoglobulins or antibody-drug conjugates. As a result, the selection of complimentary drug- and carrier properties for liposomal formulations, and the development of therapeutic combinations that integrate nanoparticulates such as liposomes, remains largely empirical. Nonetheless, there exists a growing number of examples of the successful progression of oncology drugs formulated in lipidic nanocarriers to advanced clinical development or approval. Often, the extended plasma half-life and sustained release characteristics of liposomal formulations improves the overall therapeutic index, usually because of a reduction in toxicity, but occasionally improvements in antitumor efficacy are observed. It is expected that more examples of increased antitumor efficacy may be obtained as clinical scientists learn how to optimize multi-drug therapies that exploit the unique properties of nanoparticulate drug carriers in combination with conventional or novel therapeutic agents. Further enhancements in antitumor activity and target cell selectivity may be achieved in the future as ligand-targeted lipidic nanocarriers make their way through preclinical and clinical testing.

Advances continue to be made in liposomal formulation development and optimization of their performance in specific clinical applications. With a growing understanding of the physicochemical and pharmacological factors that dictate pharmacokinetics and pharmacodynamics of liposome-incorporated oncology drugs, it is possible to envisage quantitative PK/PD modeling and simulation as a fundamental tool for (i) designing formulations having the desired pharmacological properties for specific applications; (ii) quantifying primary pharmacological and physiological processes controlling their disposition, biodistribution, and rate of delivery at the site of action; (iii) providing insights into the determinants of efficacy and safety that are operant *in vivo*; and (iv) assisting translational research that bridges data from preclinical animal models to anticipate performance in clinical settings. A recent example of how computational systems analysis can provide insights into the species scalability of oncology drug delivery from preclinical mouse models to human clinical performance [158] was able to derive and compare the hypothesized key parameters that govern liposomal drug delivery to target cells and overall efficacy at both mouse and human scales. This approach represents a significant advance. However, these and other systems analyses will be advanced further if the essential data underlying key determinants of liposome performance are obtained systematically during preclinical and clinical development.

Whereas regulatory guidance for industry on liposomal drug formulations provides recommendations on the chemistry, manufacturing controls, human PK and bioavailability, and labeling documentation for liposome drug products submitted in new drug applications (NDA) [191], there has been relatively little emphasis upon the use of computational tools that could streamline the development of novel liposomal formulations or provide a better means to improve the PK and PD characteristics of existing formulations. Mathematical models are economical and flexible by reason of their scalable parameters. They can be adapted continually and modified or extended to new circumstances as our understanding of the biology of the system and the pharmacology of the carrier formulation increases in detail. Indeed, with the aid of computational model simulations of drug delivery to tumors, and the downstream pharmacodynamics that result, it is possible to imagine that drug/carrier formulations might be designed and optimized for clinical use based on computational models capturing key performance parameters, much as the design and refinement of drugs depends heavily upon computational approaches.
